# Efficacy of denervation for osteoarthritis in the proximal interphalangeal joint (DOP): protocol of a randomized controlled trial

**DOI:** 10.1186/s13063-024-08399-w

**Published:** 2024-08-22

**Authors:** Elin M. Swärd, Jonas Beckman, Farnoush Tabaroj, Maria K. Wilcke

**Affiliations:** 1 Department of Clinical Science and Education Södersjukhuset, Karolinska Institutet, Sjukhusbacken 10, Stockholm, 11883 Sweden; 2 Department for Hand Surgery, Södersjukhuset, Stockholm, Sweden; 3 Handkirurgiska kliniken Södersjukhuset, Sjukhusbacken 10, Stockholm, 11883 Sweden

**Keywords:** Osteoarthritis, Proximal interphalangeal joint, Exercise therapy, Patient education, Denervation, Randomized controlled trial

## Abstract

**Background:**

Osteoarthritis (OA) contributes increasingly to disability worldwide. There is ample high-quality research on the treatment of knee and hip OA, whereas research on surgical and non-surgical treatment in hand OA is sparse. Limited evidence suggests that education and exercise may improve pain, function, stiffness, and grip strength in hand OA. The established surgical options in hand OA have disadvantages. Prostheses preserve motion but have a high complication rate, whereas fusions decrease function due to limited movement. There is an unmet need for high-quality research on treatment options for hand OA and a need for the development of effective and safe movement-sparing therapies.

This study aims to compare the effects of a motion-preserving surgical treatment (denervation of the proximal interphalangeal (PIP) joint) with a patient education and exercise program on patient-reported outcomes and objective function in painful PIP OA.

**Methods:**

In this parallel-group, two-armed, randomized, controlled superiority trial (RCT), 90 participants are assigned to surgical PIP joint denervation or education and exercise. Pain on load 1 year after intervention is the primary outcome measure. Secondary outcome measures include pain at rest, Patient-Rated Wrist and Hand Evaluation (PRWHE), HQ8 score, EQ5D-5L, objective physical function, complications, two-point discrimination, Mini Sollerman, consumption of analgesics, and the need for further surgery. Assessments are performed at baseline, 3 and 6 months, and 1 year after intervention.

**Discussion:**

There are no previous RCTs comparing surgical and non-surgical treatment in PIP OA. If patient education plus exercise or PIP denervation improve function, these treatments could be implemented as first-line treatment options in PIP OA. However, if denervation does not achieve better results than non-surgical treatment, it is not justified to use in PIP OA.

**Trial registration:**

Prospectively registered in ClinicalTrials.gov (NCT05980793) on 8 August 2023. URL https://classic.clinicaltrials.gov/ct2/show/NCT05980793.

**Supplementary Information:**

The online version contains supplementary material available at 10.1186/s13063-024-08399-w.

## Background

Osteoarthritis (OA) in the finger joints is very common, especially from middle age and in women, and may cause significant disability. The prevalence of symptomatic hand OA is 26% in women over 70 years of age, with an estimated lifetime risk of OA of 47% among women as compared to 25% in men [1, 2].

Common surgical options for proximal interphalangeal (PIP) joint OA are arthroplasty or arthrodesis. Arthroplasty preserves some motion [3] but has a reported complication rate of 35% 2 years postoperatively [4] and with a complication rate considerably higher than for arthrodesis [3]. A successful fusion is achieved for PIP joint arthrodesis in 95–97% of cases [5, 6] but results in impaired function due to loss of movement. Considering the disadvantages of previously described surgical treatments, there is an unmet need for effective and safe movement-sparing therapies for PIP joint OA.

Surgical joint denervation is a motion-sparing intervention, where sensory articular branches to the joint are sectioned. It has predominately been used in wrist OA, with reported high participant satisfaction, increased grip strength, and improved average pain scores [7]. Case series of PIP joint denervation in 11–54 joints have reported improved pain VAS (0-10) scores of 6–6.4 units [8–10] and an increase in range of motion of 10–25° [8, 9]. A recent systematic review of PIP joint denervation performed through a palmar approach reported 90% participant satisfaction; however, complications were observed in 14%, mainly with transient paresthesia [11]. Hence, there are low-quality evidence of encouraging results after PIP joint denervation, but to our knowledge, there are no randomized controlled studies of PIP joint denervation compared to other types of treatment.

Conservative treatment of finger joint OA includes patient education, exercise, splints, analgesics, or intraarticular glucocorticoid injections. Although patient education and exercise are considered the standard first-line treatments in hip and knee and OA [12], research regarding the effectiveness of these interventions in hand OA is limited. Reports suggest that hand exercise may improve pain and function, joint stiffness, and grip strength [13] and that education and training in ergonomic principles and joint protection also may be effective [14].

## Objective

The objective of this study is to evaluate the effects of PIP denervation compared with a nonsurgical treatment patient education and exercise on pain at rest and on load, hand function, quality of life, range of motion, and strength in patients with painful PIP joint OA.

We hypothesize that denervation will decrease pain and improve objective function more effectively than patient education plus exercise for OA in the PIP joint.

## Methods and analysis

### Study design

**Fig. 1:**
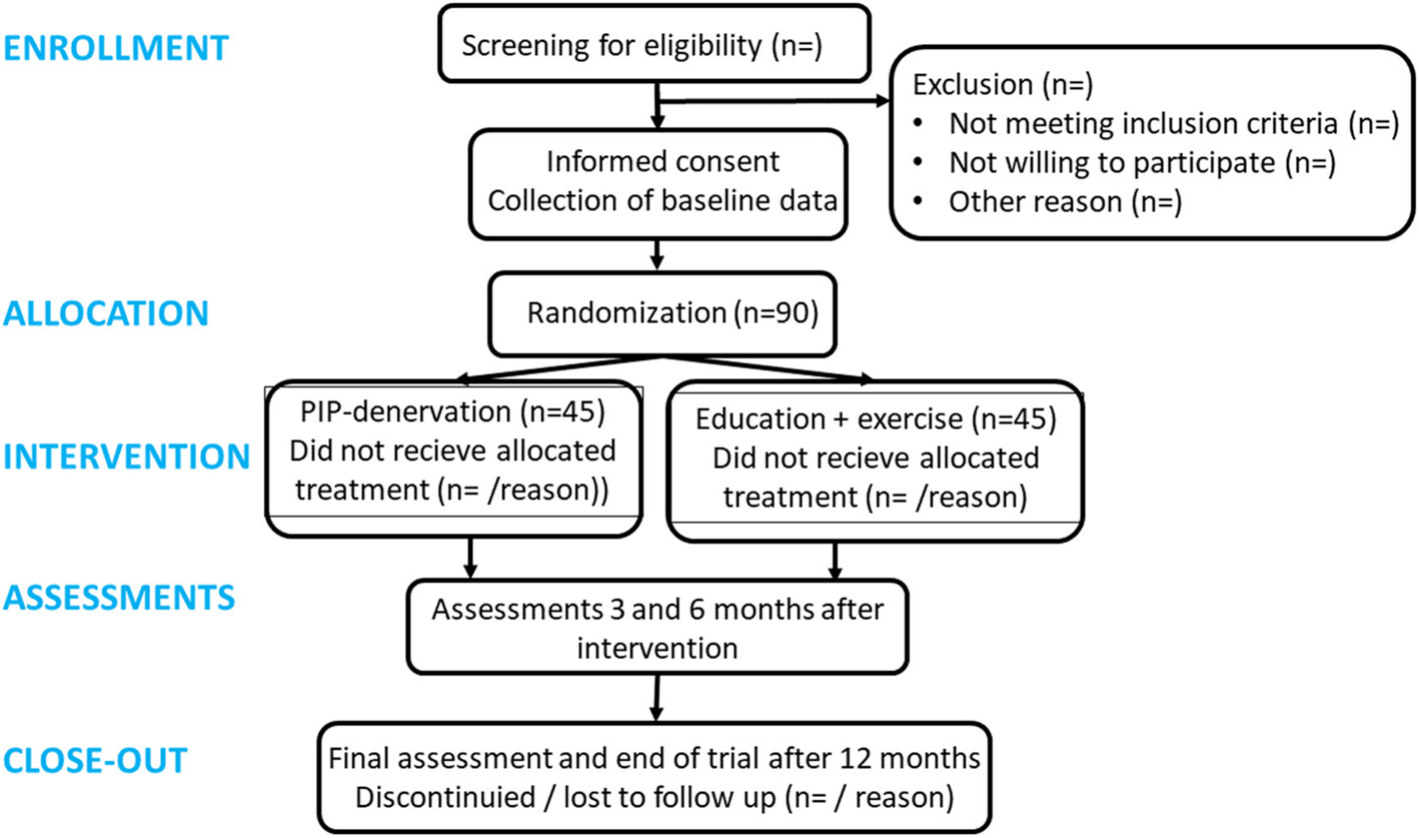
Study flowchart

**Fig. 2:**
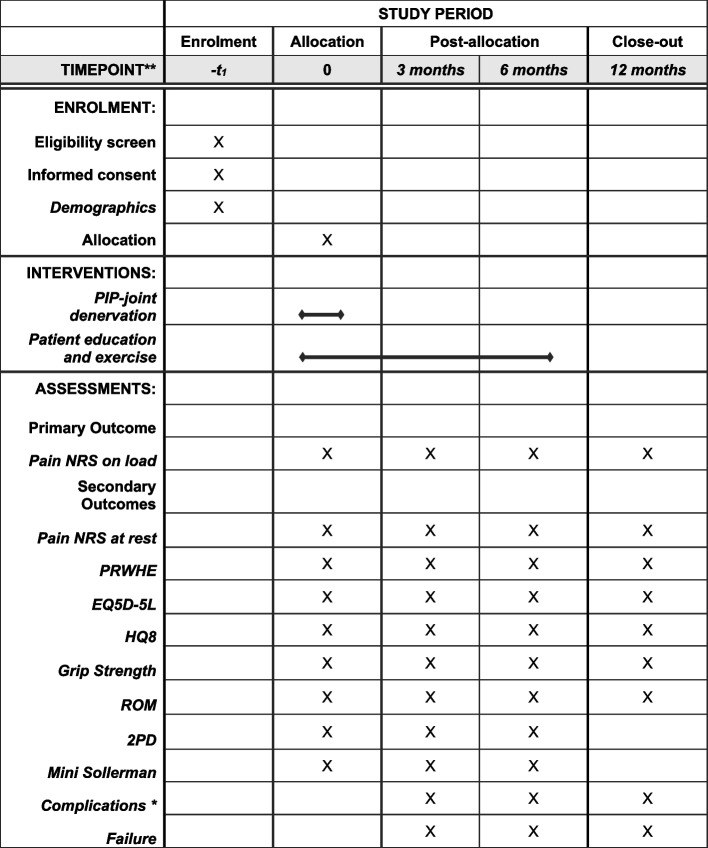
Schedule of enrolment, interventions, and assessments. Complications in the surgical group will also be assessed at suture removal 2 weeks postoperatively

The study is designed as a parallel group, two-arm, prospectively registered randomized, controlled superiority trial. Data analysts are blinded, but not participants or assessors. The trial design complies to the Standard Protocol Items: Recommendations for Interventional Trials (SPIRIT) [15] and Consolidated Standards of Reporting Trials (CONSORT) [16] guidelines. An outline of the trial is found in the study flowchart (Fig. 1) and the SPIRIT figure (Fig. 2). The SPIRIT checklist is provided as a supplemental online material (Additional file 1).

### Study setting

The study is conducted at the Department of Hand Surgery, Södersjukhuset, Stockholm, Sweden. The Department of Hand Surgery at Södersjukhuset is an accredited hand trauma and replantation center and the secondary referral center for hand surgery in the Stockholm, Södermanland, and Gotland regions, serving a population of approximately 2.8 million inhabitants (SCB Statistical database).

### Eligibility criteria

All eligible participants diagnosed with painful PIP joint OA at the Department of Hand Surgery at Södersjukhuset, Stockholm, are asked to participate in the study. One joint is included and in cases of several affected PIP joints the most symptomatic joint, according to the patient, is included.

Participants are selected based on the following eligibility criteria.

Inclusion criteria:

- Age ≥ 18 years
- Chronic (≥ 6 months) painful idiopathic or posttraumatic OA in the PIP joint
- Radiological signs of OA on posteroanterior and lateral radiographs assessed by the Kellgren–Lawrence classification (grades II–IV) [17]
- Any clinical sign of PIP joint OA: pain at palpation, pain during provocation, bony enlargement, or soft tissue swelling

Exclusion criteria:

- Rheumatoid arthritis or other chronic inflammatory arthritis. History of psoriasis affecting hand joints, gout, or pseudogout of the hand
- Ongoing infection in the hand or wrist
- Inability to co-operate with the follow-up protocol (language difficulties, severe psychiatric disorder, cognitive impairment, drug addiction)
- Systemic or Intra-articular glucocorticoids, intraarticular platelet-rich plasma, or hyaluronic acid injections in the affected joint within 3 months before enrollment

### Randomization, allocation, and blinding

All hand surgeons (> 20) working at the Department of Hand Surgery at Södersjukhuset may enroll participants. The hand surgeon in charge of the participant’s treatment collects informed consent. On the day of enrollment, a dedicated research nurse randomly assigns participants (1:1) to one of the treatment arms using sealed, unnumbered envelopes. A block randomization scheme with a fixed block size of 10 is used. The envelopes containing the allocation slips are prepared and mixed by the primary investigator. Participants are stratified according to sex, since idiopathic OA may be more common in women and men are more likely to have posttraumatic OA than women [18]. Blinding of participants and assessors is considered unfeasible due to a scar on an operated finger. Data analysts are blinded to treatment allocation (anonymized data sheets, with treatment arms coded as A or B by an external part before analyses). Participants in the non-operative treatment group are offered denervation surgery after the study’s primary endpoint (12 months). Cross-over during the study period is not allowed.

### Baseline assessment including OA classification

Demographic data for age, sex, profession, hand dominance, and medical history including previous surgeries to the hand are collected at the baseline assessment. All participants have a radiograph (posterior-anterior and lateral view) of the affected finger before inclusion. Radiographs taken within 6 months of inclusion are eligible. OA is classified according to the Kellgren–Lawrence classification, which grades OA based on osteophyte formation, narrowing of joint space, and sclerosis of the subchondral bone [17]. Grade 0 represents a normal radiograph, whereas 5 depicts a joint with large osteophytes, a severe narrowing of joint space, marked sclerosis, and certain bony deformity.

### Interventions

#### Treatment arm 1: PIP denervation

Surgical PIP-denervation is performed under local anesthesia (blood-less field with finger-ring or wide-awake local anesthesia no tourniquet (WALANT) according to the surgeon’s preference). A volar approach to the PIP joint is used and a 360-degree denervation is performed as described by Jiménez et al. [9]. The skin is closed by resorbable or non-resorbable sutures depending on the surgeons’ preferences. A soft dressing is applied and removed by the participant after 5–7 days. At 2 weeks postoperatively, a nurse removes non-resorbable sutures and examines the wound for signs of infection or stiffness. Unlimited active motion of the operated joint is encouraged immediately after surgery as tolerated by the participant. No formal hand therapy protocol is used, and participants do not receive any specific conservative treatment (orthoses, education, exercise) after surgery. However, if the finger is considerably stiff or swollen at suture removal, the participant is referred to the hand rehabilitation unit to get standard treatment for postoperative finger stiffness. The need for additional rehabilitation sessions is documented in the study notes.

#### Treatment arm 2: Patient education and exercise program

The education and exercise program follows a clinical routine practice protocol developed by the Department of Hand Surgery at Södersjukhuset in Stockholm. This specific protocol has not been evaluated in a clinical trial, but both the education and exercises are very similar to a previously published protocol in hand OA [19].

#### Education program

At study start, participants receive one 30-min face-to-face session containing oral and written information about the disease, its course and symptoms, treatment options, and self-management principles including joint protection. The information booklet (in Swedish) with general information and exercises is available as Additional file 2.

#### Exercise

**Fig. 3:**
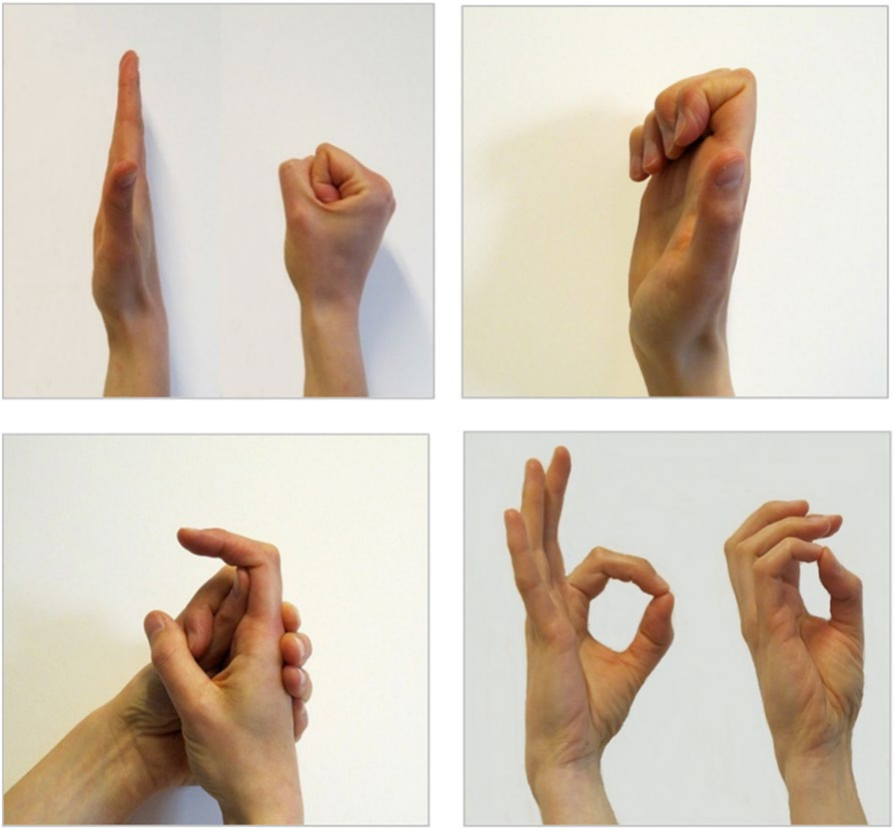
Motion-promoting exercises

**Fig. 4:**
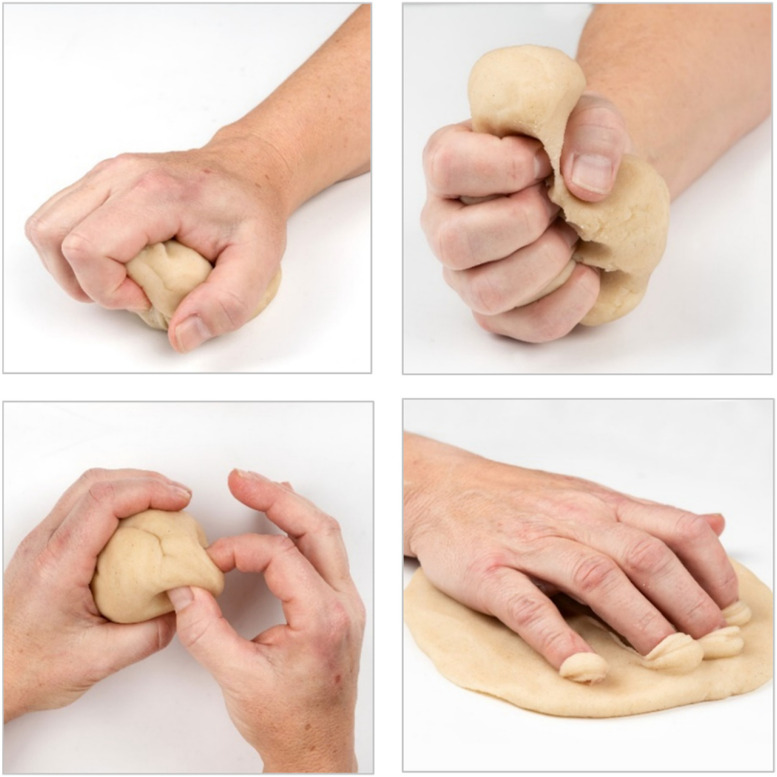
Strength-promoting exercises

The exercise program consists of four face-to-face or video sessions and one optional session (see below). Between sessions, participants are instructed to perform four motion-promoting exercises (Fig. 3) and four strength-promoting exercises (Fig. 4) at home three times a week with 10 repetitions for each exercise.

The unloaded active motion exercises (Fig. 3) promote isolated flexion/extension of PIP (Fig. 3c), global flexion/extension of the finger (Fig. 3a, b), and opposition to thumb (Fig. 3d). The participant is encouraged to hold for 3–5 s at end position.

- At study start: one face-to-face rehab session + information booklet including exercises to improve function and strength and reduce pain
- 2–4 weeks after study start: one 30-min rehab session, face-to-face or video (follow-up, modification of exercises if the participant is unable to perform all exercises due to pain or other problems)
- 3 months after study start: one 30-min rehab session, face-to-face (follow-up, modification of exercises if needed)
- 4–5 months after study start: one optional rehab session if needed, face-to-face or video (follow-up, modification of exercises if needed)
- 6 months after study start: one 30-min rehab session, face-to-face (follow-up, modification of exercises if needed)

### Concomitant care

Participants may use paracetamol or non-steroid anti-inflammatory drugs (NSAID) as needed during follow-up. Systemic or intra-articular glucocorticoids or intraarticular platelet-rich plasma (PRP) are not allowed within 3 months prior to a study assessment. The wash-out period of 3 months is based on clinical experience and evidence that the effects of PRP and steroid injections seem to wear off after 3 months [20].

### Outcome measures

Validated Swedish versions of all patient-reported outcome measures (PROM) are used. Grip strength, range of motion (ROM), static two-point discrimination (S2PD), and Mini Sollerman test are measured according to the Swedish standard for assessment of hand and upper limb measurements developed by the Swedish Healthcare Quality Registry for hand surgery (HAKIR). PROMs are collected at baseline, 3 and 6 months, and 1 year after intervention. Objective physical variables are assessed at baseline, at 3 and 6 months, and 1 year. S2PD and Mini Sollerman are tested at baseline and 3 and 6 months after intervention. Baseline assessments and measurements at 3 and 6 months and 1 year after intervention are performed by an experienced physiotherapist or occupational therapist.

### Primary outcome measure

The primary outcome measure is as follows: pain on load in the included finger (as measured by the pain numerical rating scale (NRS), item 1 in the HAKIR eight-item participant questionnaire (HQ8) score, see below) 1 year after the intervention.

### Secondary outcomes measures

#### Pain numerical rating scale (PNRS) at rest

PNRS is a numeric 11-point box scale. In this study, we use the NRS scale from HQ8 (see below) where each box represents numbers ranging from 0, 10, 20…up to 100 points. 0 corresponds to no pain and 100 the worst imaginable pain. The participants select a value that describes the perceived pain intensity in the included finger during the past week. Pain NRS is proven a valid and reliable pain scale in chronic musculoskeletal pain [21] and has a reported minimal clinically important difference (MCID) of 2 on a 0–10-point scale [22]. Finger pain on load is the primary outcome measure of this study, whereas finger pain at rest is a secondary outcome measure.

#### Patient-Rated Wrist and Hand Evaluation (PRWHE)

PRWHE [23] is a hand and wrist-specific PROM designed to evaluate pain and disability in daily living during the past week. The items are divided into a pain subscale (5 items) and a function subscale (10 items). Each item is coded from 0–10 points, and the total score ranges from 0 to 100. In calculation of the total score, pain and function scales are weighted equally. A higher PRWHE score indicates a worse disability. PRWHE is available in 21 languages and is recognized as a reliable and valid tool in patients with different wrist and hand injuries [24]. The suggested MCID of PRWHE is 14 [25].

#### The 8-item participant questionnaire (HQ8)

HQ8 is a PROM specifically developed for Swedish healthcare quality registry for hand surgery (HAKIR) [26]. It comprises 8 items evaluating pain on load, pain on motion without load, pain at rest, stiffness, weakness, numbness, cold sensitivity, and ability to perform daily activities during the past week in the hand/arm that has been operated on. Each item is graded on a numeric 11-point box scale (NRS-11) (0, 10…100). It is a single item questionnaire (a total score is not calculated). HQ8 has been shown to be reliable in both elective and traumatic hand conditions [26]. A MCID for HQ-8 has not been estimated.

#### EuroQol 5 dimensions 5 levels (EQ5D-5L)

EQ5D-5L [27] is one of the most common generic measurements of health-related quality of life and demonstrates excellent psychometric properties in different populations, conditions, and settings [28]. It comprises a visual analogue scale and a 5-item questionnaire regarding mobility, self-care, usual activities, pain/discomfort, and anxiety/depression. The response to each question is graded as the following: no, slight, moderate, severe, or extreme problems. A summary index value ranging from 0 to 1 is computed, where 0 represents death and 1 full health. Population reference data for Sweden is available [29]. The MCID of EQ5D-5L is sparsely studied in hand and wrist surgery but has been reported as 0.09 in carpal tunnel syndrome [30].

#### Grip strength and range of motion (ROM)

Grip strength is measured with a hydraulic hand dynamometer (BL5001, B&L Engineering®, Santa Ana, CA, USA). Three measurements at maximal grip are performed, and the mean in kilograms (kg) is calculated. ROM of the metacarpophalangeal (MCP), PIP, and distal inter phalangeal (DIP) joints are measured according to the “National manual for measuring motion and strength in the elbow, forearm and hand” (www.hakir.se) using a short-armed goniometer. Measurements are rounded up or down to the nearest 5° interval.

#### Mini Sollerman test

The Mini Sollerman test evaluates sensory function by measuring dexterity [31]. It is a shortened 3-task version of the original 20-task Sollerman test [32]. It comprises Sollerman tasks 4 (pick up coins from an open purse and put on the table), 8 (pick up screw-nuts and put them on the fitting bolts), and 12 (button four buttons with one hand). Each task is graded from 0 (the task could not be performed at all) to 4 (without difficulty, within 20 s and with the recommended hand grip) with a maximum total score of 12 points. To our knowledge, an MCID for Mini Sollerman has not been estimated.

#### Static two-point discrimination (S2PD)

S2PD measured by the Dellon-Mckinnon Disk-Criminator™ evaluates sensory function (discriminative touch) in the fingertips [33]. It measures the smallest distance in millimeters where a person is able sense the difference between two points of touch. S2PD is measured according to the “National assessment manual for assessment of hand function after nerve repair” (www.hakir.se).

#### Complications to treatment

All suspected complications to treatment will be recorded at the follow-up visits. Specifically, the presence of superficial or deep wound infections or loss of sensation will be recorded. S2PD and Mini Sollerman will be measured preoperatively, 3 and 6 months postoperatively to evaluate any differences in sensation before versus after treatment.

#### Need for analgesics

Consumption of paracetamol, NSAIDs, or opioids during follow-up will be recorded as a binary variable: Current pain medication, YES/NO. If yes: Type of medication and dose/day.

#### Failure

A hand surgeon performs clinical evaluations of all patients 3, 6, and 12 months after inclusion. The need for additional treatments is discussed at these appointments based on the patient’s symptoms and wishes. Failure of treatment is defined as the need for further surgery. The reason for additional surgery is noted. The date when a participant is enrolled on the waiting list for additional surgery is recorded, and the survival in months will be determined.

#### Sample size

SPSS version 29 was used for the sample size calculation. The reported MCID of NRS is 20 on a 100-point scale [22]. To our knowledge, the standard deviation (SD) of changes in pain scores in patients with PIP OA have not been previously reported. Hence, the SD is based on an estimation. SD will be calculated at the interim analysis, and the power calculation will be modified if necessary. To show a difference of 20 points in PNRS on load between intervention groups (estimated SD 30) after 12 months, 37 participants are required in each treatment arm. The power will be 80% (*p* < 0.05). To account for non-parametric outcome and loss to follow-up, we aim to include 90 participants in total, 45 in each treatment arm. Participants who withdraw from the trial before intervention will be replaced.

### Data analyses

The data analyses plan was developed in cooperation with a medical statistician at the Department for Clinical Science and Education, Karolinska Institutet (KI SÖS). Analyses will be performed using an intention-to-treat approach. In addition, per-protocol analysis will be conducted for the primary outcome as sensitivity analysis per the actual treatment received. PROMs will be reported as median (interquartile range (IQR)) and continuous variables as mean (SD). For comparison of the differences between PIP denervation versus patient education and exercise, *T*-test will be used for continuous data with normal distribution, and chi-square tests will be used for categorical data. For non-normally distributed continuous data and ordinal data, the Wilcoxon signed rank tests will be used for within-patient comparisons and rank sum tests for between-patient comparisons. Normality will be assessed by QQ-plots. No subgroup or adjusted analyses are planned. Since there are only two treatment groups, any adjustment for multiple testing is not planned for the primary outcome. Significances of secondary outcomes will be adjusted using the Holm-Bonferroni method to control for multiple significance. Missing data will not be imputed.

The level of significance is set at *p* < 0.05. Demographic data and PROMs from dropouts will be described and compared to the participants that completed the trial. Interim analyses will be performed after 20 and 40 included participants. Data will be analyzed by R or SPSS.

### Withdrawal and safety

In accordance with the ethical permit, participants may withdraw from the trial at any time without giving a reason for doing so and without any negative consequences for their future treatment. PIP denervation has a reported low frequency of complications compared to other surgical options for PIP OA. The most reported complication is transient paresthesia [11]. All surgical procedures induce a low risk of infection. Other than that, the participants are not exposed to any risks. All expected and unexpected complications to treatment are managed at the Department for Hand Surgery at Södersjukhuset. The presence of complications is assessed at the follow-up visits and interim analyses are performed after the inclusion of 20 and 40 participants to assess the frequency of complications. Participants are also provided with contact information to the Department of Hand Surgery and the research team, should any spontaneous adverse event occur. If a high frequency of unexpected complications is noted, the primary investigator will consider early termination of the study. Research participants are covered by Swedish patient insurance (LÖF).

### Retention plan

Participants in both treatment arms are scheduled for regular follow-up visits to promote adherence. Phone numbers and e-mail addresses to the researchers in charge of the trial are provided, should any questions arise. Participants who are unwilling to attend the physical follow-up assessments are offered video meetings instead. Those who do not wish to attend physical or video examinations are asked to fill in the PROMS and send them by mail. Participants that do not show up at scheduled meetings are contacted by telephone and/or mail to be rescheduled for a new appointment. All appointments after enrollment are free of charge. No reimbursements for travel expenses or other costs are provided. In case of dropouts, the reasons for dropping out will be recorded, and the data gathered up to that point will be collected and analyzed.

### Data management and confidentiality

The research team is responsible for trial coordination, conduct, and data management. Data is handled in accordance with the General Data Protection Regulation (GDPR). Data will be transferred from paper-based PROMS to protected electronic databases at Södersjukhuset and Karolinska Institutet. A member of the research group will cross-check the transferred data for errors at interim analyses and before the final analyses. Participants are given an anonymized serial number. The key to the anonymous serial numbers will be kept in protected databases at Södersjukhuset and Karolinska Institutet. Only researchers responsible for the trial have access to the data files and the decoding key. All data will be analyzed anonymized, and results will be presented at a group level. There is no formal data monitoring committee, but the research team will monitor data during interim analyses.

## Discussion

OA is a widespread disease that contributes substantially to years lived with disability. Worldwide, 595 million people were estimated to have symptomatic radiologically confirmed OA in 2020 and the number of cases is rising [34]. Even though hand joints alongside knee and hip joints are most commonly affected by OA, there is a lack of high-quality evidence regarding the effectiveness of treatments in hand OA. To our knowledge, this is the first randomized controlled trial comparing a surgical to a non-surgical treatment in PIP-OA and the first randomized controlled evaluation of PIP denervation.

The global prevalence of symptomatic hand OA is expected to rise by almost 50% from 2020 to 2050 [34]. Hence, there is a need for development of simple, affordable, and cost-effective treatments in OA. Prostheses are expensive and require specialized hand rehabilitation, whereas arthrodesis demands prolonged immobilization. PIP joint denervation is a simple procedure where specialized rehabilitation is not needed. Hence, PIP denervation may be more cost-effective in PIP OA treatment and offer a feasible treatment option also in low/middle-income countries where access to specialized hand rehabilitation and prosthesis surgery is limited.

Patient education and exercise is chosen as the comparator since it is the standard first-line treatment in all types of OA [12]. Another option could be to compare against placebo (sham surgery). However, a recent systematic review and meta-analysis on the use of placebo and nonoperative controls (no treatment, usual care, or exercise program) in surgical trials conclude that placebo-controlled surgical trials may be unnecessary since sham surgery does not seem to have a greater placebo effect than non-operative controls [35]. Therefore, we argue that it is more efficient and ethical to use patient education and exercise as a comparator instead of subjecting research subjects to sham surgery.

Pain in the included finger, measured as PNRS, was chosen as primary outcome measure for several reasons. Firstly, the main goal of OA treatment in clinical practice is to reduce pain. Secondly, several previous studies of PIP denervation have used pain (VAS) as primary outcome [8–10]. We chose PNRS instead of VAS since it is part of the HQ8 questionnaire, and we wanted to lower participant response burden. NRS and VAS scores are reported to correspond well in a postoperative setting, making comparisons between studies possible [36]. Thirdly, pain NRS is proven a valid and reliable pain scale in chronic musculoskeletal pain [21].

A potential limitation of this study is that the exercise program addresses the whole hand, whereas denervation only affects one finger joint. Hence, the training program may hypothetically improve region-specific PROMs such as PRWHE more in cases with several affected finger joints. However, multiple finger OA is unlikely to affect the primary outcome PNRS, since the PNRS only evaluates pain in the included finger. Another limitation is the relatively short follow-up, and if PIP denervation is effective, further trials with long-term follow-up are warranted. An additional limitation is the lack of blinding of participants and assessors, possibly introducing biases to measurements [37]. Blinding of participants is not considered possible due to surgical/non-surgical treatment arms. A blinding of assessors (by application of a glove or adhesive plaster) may negatively affect ROM or sensation, leading to systematic measurement errors. Measurements are performed by an occupational or physiotherapist to reduce possible observer or measurement bias. The major strengths of this study compared to previous research is the RCT design, a large sample size, and a pragmatic design, enhancing generalizability.

If PIP denervation significantly relieves pain and improves function, it may offer an important treatment option since it preserves motion, has few reported complications, does not require immobilization, and is technically easier than other surgical options. However, since all surgical procedures come with a risk of complications, PIP denervation should not be used for PIP joint osteoarthritis if it does not provide better outcomes than non-surgical treatment.

## Trial status

This is protocol version number 2.0 (from the 14 December 2023). No additional changes have been made hereafter. Any future important protocol modifications require permission from the ethical review board. Recruitment began on 19 September 2023 and recruitment is estimated to be completed in 2027.

### Supplementary Information

Additional file 1: SPIRIT Checklist for *Trials*.

Additional file 2: Information booklet

## Data Availability

Access to medical data on individual participants is restricted by Swedish law in the General Data Protection Regulation (GDPR) (https://www.imy.se/en/organisations/data-protection/) Hence, the datasets of this study will not be publicly available. Data can be made be available for researchers after application to and approval by the Swedish Ethical Review Authority and the local data safety committee at Södersjukhuset (GDPR.sodersjukhuset@regionstockholm.se).

## Abbreviations

CONSORT: Consolidated Standards of Reporting Trials
DIP joint: Distal interphalangeal joint
EQ5D-5L: EuroQol 5 Dimensions 5 levels
GDPR: General Data Protection Regulation
GEE: Generalized estimating equations
HAKIR: Swedish Healthcare Quality Registry for hand surgery
HQ8: HAKIR eight-item participant questionnaire
IQR: Interquartile range
MCID: Minimal clinically important difference
MCP joint: Metacarpophalangeal joint
NSAID: Non-steroid anti-inflammatory drug
OA: Osteoarthritis
PNRS: Pain numerical rating scale
PIP joint: Proximal interphalangeal joint
PROM: Patient-reported outcome measure
PRP: Platelet-rich plasma
PRWHE: Patient-Rated Wrist and Hand Evaluation
RCT: Randomized controlled trials
ROM: Range of motion
SD: Standard deviation
S2PD: Static two-point discrimination
SPIRIT: Standard Protocol Items: Recommendations for Interventional Trials
WALANT: Wide-awake local anesthesia no tourniquet
