# Supplementary material for: Efficacy of denervation for osteoarthritis in the proximal interphalangeal joint (DOP): protocol of a randomized controlled trial
Source: Trials. 2024 Aug 22;25:553. doi: 10.1186/s13063-024-08399-w (PMC11340183; doi:10.1186/s13063-024-08399-w)
Supplement: Supplementary file 2 — Additional file 2: Information booklet [file 13063_2024_8399_MOESM2_ESM.pdf]

Patientinformation

Fingerledsartros

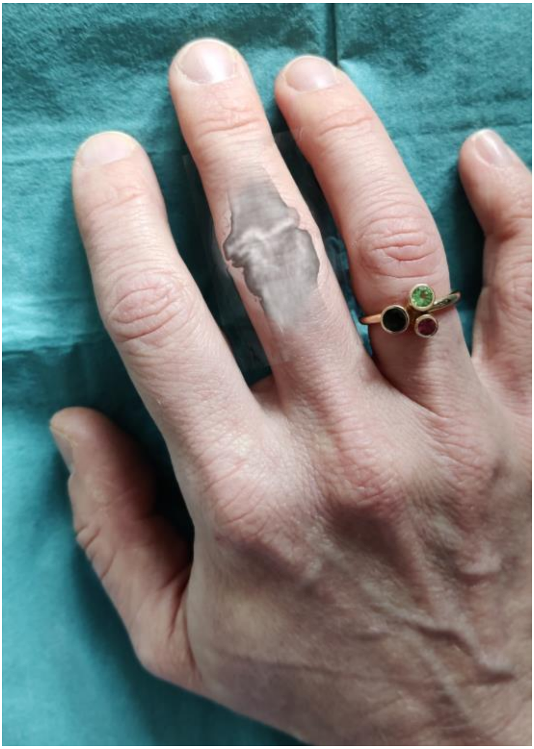

SÖDERSJUKHUSET

Vad är Fingerledsartros?

Artros är en av de vanligaste kroniska sjukdomarna i världen. Sjukdomen drabbar kroppens leder och innebär att ledens brosk, ben och omgivande mjukdelar bryts ner. I en frisk led finns en balans mellan uppbyggnad och nedbrytning av ledbrosket, men i en led som drabbats av artros uppstår en obalans där de nedbrytande faktorerna är större än de uppbyggande. I och med att ledbrosket bryts ner möts ben mot ben i leden vilket ofta orsakar smärta. Fingerledsartros är mycket vanligt, framför allt hos kvinnor och förekomsten ökar vid stigande ålder. En eller flera fingerleder kan drabbas av artros och är allra vanligast i fingrarnas ytterleder. Artros i fingrarnas mellanleder är också vanligt förekommande.

Vilka symtom kan man få?

Man kan ha artros i fingerlederna utan att ha några symptom och besvärens omfattning kan variera över tid. Typiska symtom vid fingerledsartros är att lederna smärtar vid rörelse och belastning av handen. Ibland gör det också ont i vila. Lederna kan svullna upp, bli knotiga och ändra form. Med tiden kan lederna bli stela, vilket kan medföra att händerna kan upplevs fumliga och svaga. Det kan bli svårare att greppa om föremål och att använda handen vid belastning.

Vad är orsaken?

Orsaken till fingerledsartros är ofta ärftlig, men artrosen kan också utvecklas efter benbrott, ledbandsskador eller infektioner i leden. Ibland uppkommer artros utan någon känd bakomliggande orsak. Till skillnad från artros i höft- och knäleder beror artros i fingerleder i mindre utsträckning på livsstilsfaktorer.

Behandling

Grundbehandlingen vid artros innefattar utbildning om sjukdomen och anpassad fysisk träning. Fysisk träning är viktigt vid artros, eftersom näringstillförseln till ledens brosk är beroende av att leden

utsätts för rörelse och belastning. Regelbunden rörelse håller också omkringliggande ledband och ledkapsel flexibla, vilket minskar risken för stelhet i leden och kan även öka ledens rörlighet.

• Artrosdrabbade leder gör ibland ont vid rörelse och belastning. Viss smärta vid träning är inte farligt. Det är viktigt att fortsätta träningen även om det gör ont. Om du får långvarig smärta som inte klingar av ett dygn efter träning kan du behöva anpassa träningsprogrammet. Kontakta då din fysioterapeut eller arbetsterapeut för råd.

• Ibland kan man behöva ta smärtstillande tabletter för att kunna träna. Diskutera med din läkare vilken typ av smärtstillande behandling som kan vara aktuell i ditt fall.

• Försök att arbeta med varierande uppgifter. Undvik långa perioder av statiskt arbete t.ex. att sticka, putsa fönster, snickra eller andra aktiviteter som innebär att hålla ett och samma grepp längre stunder. Ta många korta pauser under aktiviteten. Måste du bära så fördela vikten på båda händerna. Vid behov kan kompression användas i smärtlindrande syfte.

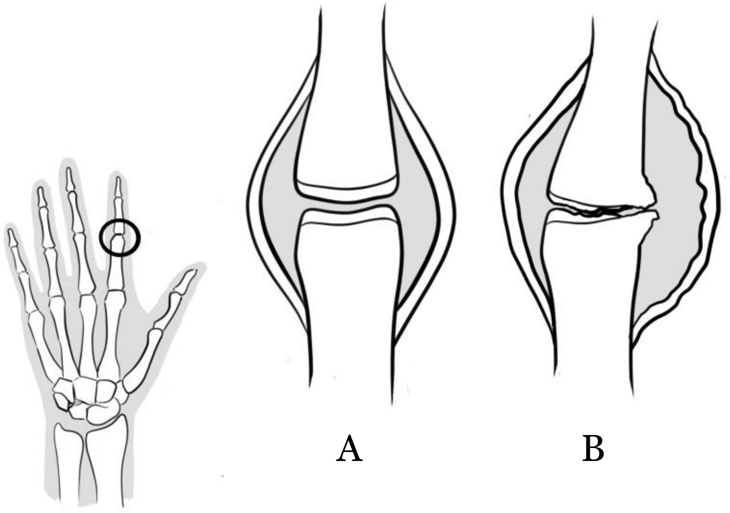

A- En frisk led med ledbrosk och ledspringa.

B- En led med artros där ledkapsel och ledbrosket börjat brytas ned, med minskad ledspringa och ben möter ben.

Träningsprogram Rörlighet

Rörelserna genomförs lugnt och koncentrerat. Det är viktigt att både knyta och sträcka i lederna. Håll kvar i rörelsens ytterläge 3-5 sekunder.

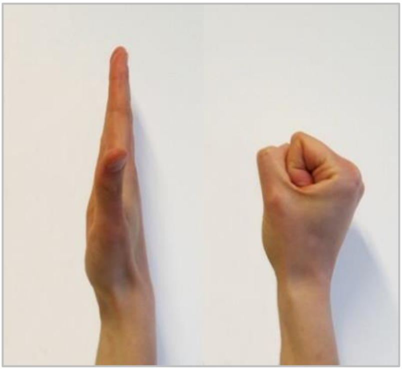

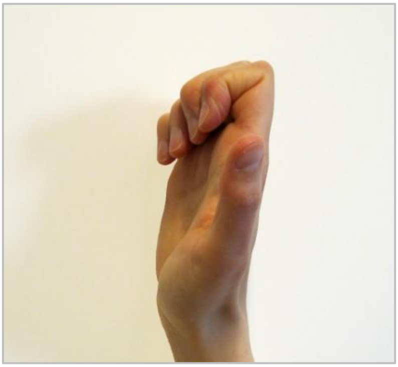

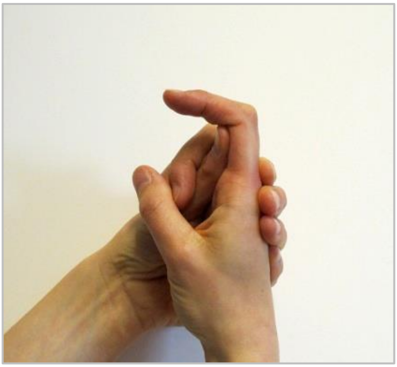

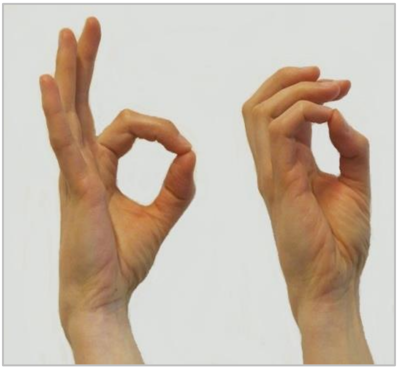

Träningsprogram Styrka

Motståndsträning med deg ger ökad styrka i handen samt belastar och näringsrätter ledbrosket. Träna lugnt och fokuserat

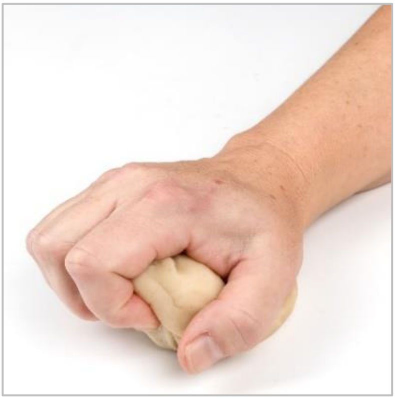
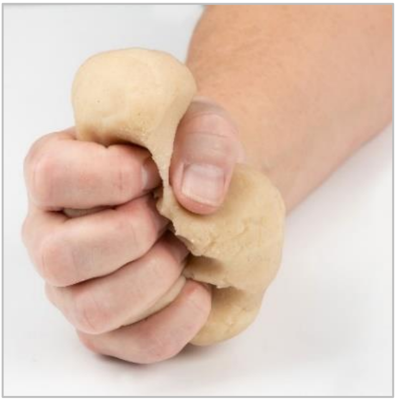
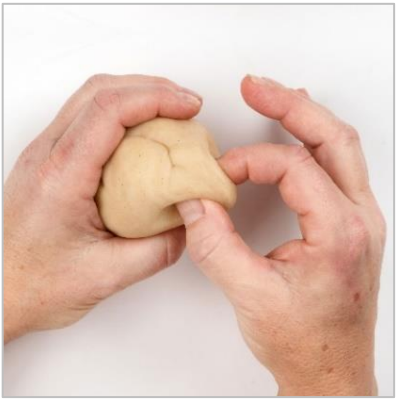
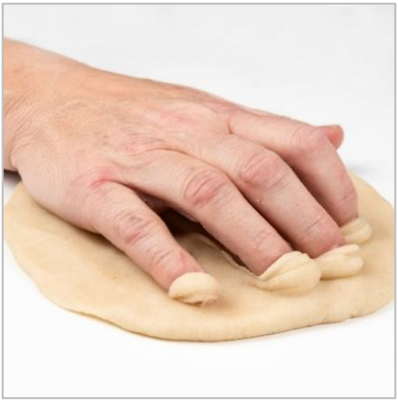

Utför träningsprogrammet, både för rörlighet och med styrka med deg

3 ggr/vecka och **10 repetitioner** per övning.

Vi är en del av Stockholms läns landsting

Vid frågor ring!

Arbetsterapeut / Fysioterapeut...

Telefon...

Kontakt

Handkirurgiska kliniken, mottagning

Kontakta oss via 1177 Vårdguidens e-tjänster. Du loggar in via www.1177.se.

Du kan också kontakta oss via telefon:
08-123 620 00, måndag - fredag.

Via 1177 Vårdguiden kan du också få rådgivning dygnet runt, telefon: 1177.

SÖS
SÖDERSJUKHUSET

Sjukhusbacken 10, 118 83 Stockholm. Telefon 08-123 610 00
SL-Buss 3, 4, 164. Pendeltåg Stockholms Södra
www.sodersjukhuset.se
